# Integrated Use of Molecular Techniques to Detect and Genetically Characterise DNA Viruses in Italian Wolves (*Canis lupus italicus*)

**DOI:** 10.3390/ani11082198

**Published:** 2021-07-24

**Authors:** Andrea Balboni, Lorenza Urbani, Mauro Delogu, Carmela Musto, Maria Cristina Fontana, Giuseppe Merialdi, Giuseppe Lucifora, Alessia Terrusi, Francesco Dondi, Mara Battilani

**Affiliations:** 1 Department of Veterinary Medical Sciences, Alma Mater Studiorum—University of Bologna, 40064 Bologna, Italy; a.balboni@unibo.it (A.B.); lorenza.urbani2@unibo.it (L.U.); mauro.delogu@unibo.it (M.D.); carmela.musto2@unibo.it (C.M.); alessia.terrusi2@unibo.it (A.T.); f.dondi@unibo.it (F.D.); 2 Istituto Zooprofilattico Sperimentale della Lombardia e dell’Emilia Romagna (IZSLER), 40127 Bologna, Italy; mariacristina.fontana@izsler.it (M.C.F.); giuseppe.merialdi@izsler.it (G.M.); 3 Istituto Zooprofilattico Sperimentale del Mezzogiorno (IZSM), 80055 Naples, Italy; giuseppe.lucifora@cert.izsmportici.it

**Keywords:** Canine adenovirus, Canine circovirus, *Carnivore protoparvovirus 1*, Italy, phylogeny, wolf

## Abstract

**Simple Summary:**

In our study, different quantitative and qualitative molecular techniques were used to detect and genetically characterise *Carnivore protoparvovirus 1*, Canine adenovirus type 1 and 2 (CAdV-1 and CAdV-2), and Canine circovirus (CanineCV) in Italian wolves (*Canis lupus italicus*) of the Italian Apennines. Carnivore protoparvoviruses were the most frequently detected viruses, followed by CanineCV and CAdV. All the wolves tested positive for at least one of the DNA viruses screened, and 47.8% of the subjects were coinfected with two or three viruses. From viral sequences analysis, close correlations emerged between the viruses identified in the wolves and those circulating in domestic dogs, suggesting that the same viruses infect wolves and domestic dogs. Further studies are needed to investigate if pathogens are transmitted between the two species.

**Abstract:**

In this study, internal organs (tongue, intestine, and spleen) of 23 free-ranging Italian wolves (*Canis lupus italicus*) found dead between 2017 and 2019 were tested for *Carnivore protoparvovirus 1*, Canine adenovirus (CAdV), and Canine circovirus (CanineCV) using real-time PCR assays. Genetic characterisation of the identified viruses was carried out by amplification, sequencing, and analysis of the complete viral genome or informative viral genes. All the wolves tested positive for at least one of the DNA viruses screened, and 11/23 were coinfected. Carnivore protoparvoviruses were the most frequently detected viruses (21/23), followed by CanineCV (11/23) and CAdV (4/23). From the analysis of the partial VP2 gene of 13 carnivore protoparvoviruses, 12 were canine parvovirus type 2b, closely related to the strains detected in dogs and wild carnivores from Italy, and one was a feline panleukopenia-like virus. Of the four CAdV identified, two were CAdV-1 and two were CAdV-2. The complete genome of seven CanineCVs was sequenced and related to the CanineCV identified in dogs, wolves, and foxes worldwide. Close correlations emerged between the viruses identified in wolves and those circulating in domestic dogs. Further studies are needed to investigate if these pathogens may be potentially cross-transmitted between the two species.

## 1. Introduction

Wild carnivores are threatened by habitat fragmentation, climate change, hunting, and loss of prey [1,2]. Infectious diseases are considered a threat, and the growth of human populations and consequent increase in domestic animals around protected areas enhance the opportunities for disease transmission among wild and domestic animals [3]. General knowledge about the disease dynamics in free-ranging carnivores is sparse, mainly due to the lack of epidemiological information concerning pathogen distribution among wild populations [2]. Wild carnivores are potentially susceptible to several viral diseases that can severely affect populations through increased mortality or decreased general health [4], such as distemper [5,6] and parvovirosis [6,7]. The *Carnivore protoparvovirus 1* species, *Parvoviridae* family, comprises the most important parvoviruses responsible for acute gastroenteritis and leukopenia in domestic and free-ranging carnivores worldwide, including wolves, such as canine parvovirus type 2 (CPV) and feline panleukopenia virus (FPV) [6,7,8,9,10]. The original CPV type 2 (CPV-2) was first reported in the late 1970s in domestic dogs [11] and originated from FPV or related viruses in wild carnivores [12]. Although CPV is a relatively new host-specific pathogen, the virus has spread rapidly in the form of the three antigenic variants 2a, 2b, and 2c (CPV-2a, CPV-2b, and CPV-2c), able to infect cats and to occasionally jump species barriers [7,13,14,15]. In Europe, the presence of CPV has been documented in a variety of free-ranging carnivore populations from canids, such as foxes and wolves [16,17,18,19,20,21], to mustelids [22,23], through serology or molecular methods. On the other hand, FPV has been reported in European wild carnivores, including badgers [16] and Egyptian mongooses [16,23]. Transmission between wild and domestic carnivores is supported by a number of molecular studies that showed, based on sequencing of the viral VP2 gene, that wild and domestic carnivores shared identical or closely related parvoviruses [23,24]. 

The genus *Mastadenovirus*, within the family *Adenoviridae*, includes two types of Canine adenoviruses (CAdV), CAdV-1 and CAdV-2, which infect dogs and cause infectious canine hepatitis (ICH) and infectious tracheobronchitis, respectively. Apart from domestic dogs, several members of the *Canidae* [25,26,27,28,29], *Ursidae* [30], and *Mustelidae* families are susceptible to CAdV-1 and can even die from infection [31]. Although few studies on CAdV-1 infection in wild carnivores were carried out, it was frequently reported in association with encephalitis [27,28,32], including in wolves. CAdV-2 has been detected in faecal samples of red foxes and wolves, but its pathogenic role in domestic dogs as well as in wild carnivores is unclear [33,34,35]. The recommended vaccination of dogs using modified live CAdV-2 confer cross-protection and has greatly reduced the circulation of CAdV-1 among domestic dogs in developed countries [36], but unvaccinated dogs in rural areas may be a source of the virus [37]. 

Canine circovirus (CanineCV) is a relatively new canine pathogen discovered within the genus Circovirus and the family *Circoviridae*. Its pathogenic role is still uncertain, but its detection was potentially associated with gastrointestinal and neurological symptoms [38,39,40,41,42]. CanineCV was first identified in serum from healthy domestic dogs in 2011 in the USA [43]. Since then, the virus has been reported in dogs worldwide [39,40,44] and in wild animals, such as wolves, foxes, and badgers [19,41,45,46]. 

The Italian wolf (*Canis lupus italicus*) population strongly declined in the past. Since the turn of the last century, wolves were confined to the south of the Italian Alps and, in the 1970s, they were reduced to approximately 100 subjects surviving in two fragmented subpopulations in the central-southern Italian Apennines [47]. The Italian wolves are presently expanding in the Apennines, and started to recolonise the western Alps in Italy, France, and Switzerland about 30 years ago [48]. Surveillance activity of the viral pathogens in wolves are useful for assessing their spread and preventing a possible effect on population size.

In this study, different quantitative and qualitative molecular techniques were used to detect and genetically characterise the DNA viruses (carnivore protoparvoviruses, CAdV-1 and 2, and CanineCV) in Italian wolves from the Italian Apennines in order to investigate the circulation of these viruses in the wolf population and to evaluate the genetic correlations with viruses circulating in domestic dogs.

## 2. Materials and Methods

### 2.1. Study Design and Sampling

Italian wolves (*Canis lupus italicus*) found dead in the Italian Apennines between 2017 and 2019 and referred to the Department of Veterinary Medical Sciences of the University of Bologna, or to the Lombardy and Emilia Romagna Experimental Zootechnic Institute, Italy, were included in the study. A complete post-mortem examination was carried out on each wolf included and their internal organs (tongue, intestine, and spleen) were sampled. Signalment data (sex, coat, geographical origin, and year of sampling) of the wolves were retrieved from registrations and age was estimated based on body size, weight, and tooth wear [49]. Wolves were classified as puppies (≤12 months of age), sub-adults (age comprised between 13 and 24 months), and adults (>24 months of age).

The presence of *Carnivore protoparvovirus 1*, CAdV, and CanineCV DNA was detected using specific quantitative molecular assays. The complete genome or informative genes of the identified viruses were amplified, sequenced, and analysed.

### 2.2. Detection of Carnivore Protoparvovirus 1, Canine Adenovirus, and Canine Circovirus DNA 

DNA extraction from the tongue, intestine, and spleen samples was performed using the NucleoSpin Tissue Kit (Macherey-Nagel, Düren, Germany) according to the manufacturer’s instructions. The extracted DNA was eluted in 100 µL of elution buffer and stored at −20 °C until use.

The detection of *Carnivore protoparvovirus 1*, CAdV and CanineCV DNA was carried out with three specific SYBR Green real-time PCR (qPCR) assays, each of which performed using the PowerUp SYBR Green Master Mix (Thermo Fisher Scientific, Life Technologies, Carlsbad, CA, USA), following the manufacturer’s instructions, in a total volume of 20 μL, and the StepOnePlus Real-Time PCR System (Thermo Fisher Scientific, Life Technologies, Carlsbad, CA, USA).

The presence of canine protoparvoviruses DNA was investigated in tongue and intestine samples by using a qPCR targeting a fragment of 99 nucleotides (nts) in the main capsid protein VP2 gene, with the primers A-for (5′- AGC TAC TAT TAT GAG ACC AGC TGA G -3′) and A-rev (5′- CCT GCT GCA ATA GGT GTT TTA A -3′) [50]. The presence of CAdV DNA was investigated in tongue and spleen samples by using a qPCR targeting a fragment of 166 nts in the E3 gene, with the primers CAdV-qPCR-For3 (5′- CTG ASA CTG CWA TRM CTA TAT AYA TTT CCA -3′) and CAdV-qPCR-Rev2 (5′- GAC ATA GAR ACR CAG GAC CCA GA -3′), and able to discriminate the two viral types (CAdV-1 and CAdV-2) on the basis of melting curve analysis [51]. The presence of CanineCV DNA was investigated in intestine and spleen samples by using a qPCR targeting a fragment of 132 nts in the intergenic region (IR) between the ends of the two major open reading frames (ORFs), with the primers CaCV 909–931 qPCR-For (5′- CTG AAA GAT AAA GGC CTC TCG CT -3′) and CaCV 1020–1040 qPCR-Rev (5′- AGG GGG GTG AAC AGG TAA ACG -3′) [46]. The thermal cycling of all reactions consisted of 95 °C for 5 min and 45 cycles of 95 °C for 15 s and 60 °C for 1 min. The melting experiment for the evaluation of the specificity of each reaction was performed after the last extension step by a continuous increment from 55 °C to 98 °C and specific melting temperatures were about 81–82 °C for carnivore protoparvoviruses, 73 °C for CAdV-1, 80 °C for CAdV-2, and 93 °C for CanineCV. Viral DNA copy number determination was carried out by absolute quantification using the standard curve method. Serial 10-fold dilutions of a plasmid (pCR4 plasmid; Thermo Fisher Scientific, Life Technologies, Carlsbad, CA, USA) containing one copy of the respective viral target sequence were used as external standards for the construction of the assay standard curve by plotting the plasmid copy number against the corresponding threshold cycle values. The limit of detection (LOD) of the reactions were determined based on the highest dilution of recombinant plasmid possible to amplify with good reproducibility and was found to be one copy/μL for carnivore protoparvoviruses, 10 copies/μL for CAdV-1 and CAdV-2, and five copies/μL for CanineCV [46,50,51]. The DNA samples and standards were repeated within each run in duplicate. A no template control, consisting of ultrapure water, underwent analysis simultaneously. Samples showing an exponential increase in the fluorescence curve, a target DNA amount greater than or equal to the LOD, and a specific melting peak in both replicates were considered positive.

### 2.3. Genetic Characterisation of the Viruses Identified

The carnivore protoparvoviruses, CAdV-1, CAdV-2, and CanineCV identified by the screening qPCRs were genetically characterised by the integrated use of end-point polymerase chain reaction (PCR) assays, sequencing, and bioinformatics analysis. For wolves that showed specific qPCR products for the same viral DNA in more than one organ, the biological matrix showing the highest amount of target DNA and no non-specific products was chosen for subsequent analyses. Each PCR was performed using the Phusion Hot Start II DNA Polymerase (Thermo Fisher Scientific, Life Technologies, Carlsbad, CA, USA), containing a high-fidelity DNA polymerase, according to the manufacturer’s instruction, in a total volume of 50 μL containing 0.5 µM of each primer, 5X HF buffer, 2.5 mM dNTP, 2 U/µL Phusion Hot Start II DNA Polymerase, and 5 µL of DNA extract. A no template control, consisting of ultrapure water, underwent analysis simultaneously.

For the identified carnivore protoparvoviruses, a fragment of the VP2 gene was amplified because, in addition to being informative for genetic characterization, it was short in length, increasing the probability of obtaining the amplicon even in samples with low amounts of viral DNA. The fragment of the VP2 gene was amplified using a hemi-nested PCR with the primers C-for (5′- CCA TTT CTA AAT TCT TTG -3′), D-rev (5′- TTT CTA GGT GCT AGT TGA G -3′) and E-rev (5′- AAG TCA GTA TCA AAT TCT T -3′), producing an amplicon of 569 nts, as reported by Balboni and colleagues [50]. The canine parvovirus 2a (CPV-2a) 791/2014 (MK348096) [52] was used as positive control.

The complete hexon and fiber genes of the identified CAdV were amplified using two PCR with the primers CAdV-Hexon-For1 (5′- GAA GTT TGC CGA CCC TGT C -3′) and CAdV-Hexon-Rev1 (5′- ACT ATG GCT CGC AGC TCT TC -3′), and CAdV-Fiber-For1 (5′- ATG TGG TCT CTC CCR ACA GC -3′) and CAdV-Fiber-Rev1 (5′- ACT TTT CCT GAA GGC GGY AG -3′), respectively, as reported by Balboni and colleagues [53]. Amplicons of 2813 nts were produced for both the CAdV-1 and CAdV-2 hexon genes, whereas amplicons of 1787 nts and 1748 nts were produced for the CAdV-1 and CAdV-2 fiber genes, respectively. The CAdV-1 417-2013-L (KP840546 and KP840547) [53] was used as positive control.

The complete genome of the identified CanineCV was amplified integrating rolling circle amplification (RCA) and PCR methods [46]. The RCA was performed on the positive samples to increase the amount of circular DNA using the TempliPhi 100 amplification kit (GE Healthcare, Chicago, IL, USA) following the manufacturer’s instructions. Subsequently, viral DNA was amplified by two PCRs with the primers CaCV 1020–1040 For (5′- CGT TTA CCT GTT CAC CCC CCT -3′) and CaCV 909-931 Rev (5′- AGC GAG AGG CCT TTA TCT TTC AG -3′), and CaCV 3′-3′ For (5′- ATG GTG GGA TGG CTA CGA TG -3′) and CaCV 3′-3′ Rev (5′- CAA GGA AGA GGG AAT GCT ACA AG -3′), respectively, as reported by De Arcangeli and collaborators [46]. The CanineCV 73/2017 (MT180081) [45] was used as positive control.

Five microlitres of each amplicon was separated by electrophoresis in a 1–2% (*w*/*v*) agarose gel stained with Midori Green Advance DNA Stain (Nippon Genetics, Chiyoda-ku, Tokyo, Japan) in 1 X Tris-acetate ethylene diamine tetra-acetic acid (TAE) buffer, together with a GeneRuler 100 bp or 1 kb DNA Ladder (Thermo Fisher Scientific, Fermentas, Waltham, MA, USA), and visualised with ultraviolet (UV) light. Amplicons of the expected size were considered positive, purified using the QIAquick PCR Purification Kit (QIAGEN, Hilden, Germany) according to the manufacturer’s instructions, and directly sequenced by Sanger method (BioFab Research, Rome, Italy). The obtained sequences were assembled, analysed with BLAST web interface (https://blast.ncbi.nlm.nih.gov/Blast.cgi, accessed on 9 April 2021), aligned with reference sequences from the GenBank database (https://www.ncbi.nlm.nih.gov/genbank/, accessed 12 April 2021, Tables S1–S3) using the ClustalW method implemented in BioEdit software version 7.2.5 (Tom Hall, Ibis Biosciences, Carlsbad, CA, USA), and translated into amino acid sequences.

Phylogeny was carried out on nucleotide sequences using MEGA X software version 10.1.7 (Pennsylvania State University, University Park, PA, USA) [54]. A phylogenetic tree of the partial VP2 gene of *Carnivore protoparvovirus 1* was constructed using the Neighbor-Joining method and Tamura 3-parameters (T92) model with a gamma distribution. A phylogenetic tree of the multiple gene sequences (concatenated hexon and fiber genes sequences) of CAdV was constructed using the Maximum Likelihood method and Hasegawa–Kishino–Yano (HKY) model with invariable sites. A phylogenetic tree of the complete genome of CanineCV was constructed using the Maximum Likelihood method and General Time Reversible (GTR) model with a gamma distribution and invariable sites. The robustness of the individual nodes on the phylogenetic trees was estimated using 1000 bootstrap replicates.

## 3. Results

### 3.1. Study Population

During the study period, 23 wolves were included. Signalment data and the year of sampling of the wolves are reported in Table 1.

### 3.2. Detection of Carnivore Protoparvovirus 1, Canine Adenovirus, and Canine Circovirus DNA 

Twenty-one out of 23 (91.3%) wolves tested positive for *Carnivore protoparvovirus 1* DNA (Table 1): eleven were positive in both analysed organs (tongue and intestine), whereas five wolves were positive only in tongue and five only in intestine samples, respectively (Table 2). The overall median quantity of *Carnivore protoparvovirus 1* DNA was 7.3 × 10^1^ copies of the target DNA per microliter of template (range: 1.4 × 10^0^–2.6 × 10^4^).

Four out of 23 (17.4%) wolves tested positive for CAdV DNA (Table 1): two were positive for CAdV-1 in spleen samples and two for CAdV-2 in tongue samples (Table 2). The overall mean quantity of CAdV-1 DNA in the spleen samples was 3.3 × 10^4^ copies of the target DNA per microliter of template (range: 5 × 10^1^–6.6 × 10^4^) and the overall mean quantity of CAdV-2 DNA in tongue samples was 1.1 x 10^1^ copies of the target DNA per microliter of template (range: 1 × 10^1^–1.2 × 10^1^).

Eleven out of 23 (47.8%) wolves tested positive for CanineCV DNA (Table 1): seven were positive in both analysed organs (intestine and spleen), whereas one and three were positive only in intestine or spleen samples, respectively (Table 2). The overall median quantity of CanineCV DNA was 6.8 × 10^2^ copies of the target DNA per microliter of template (range: 8.2 × 10^0^–3.7 × 10^7^).

All of the 23 wolves tested positive for at least one of the DNA viruses screened, and 11/23 (47.8%) were coinfected (Table 1 and Table 2): nine were positive for two viruses (eight for *Carnivore protoparvovirus 1* and CanineCV, and one for *Carnivore protoparvovirus 1* and CAdV-2), and two were positive for three viruses (one for *Carnivore protoparvovirus 1*, CAdV-2, and CanineCV, and one for *Carnivore protoparvovirus 1*, CAdV-1, and CanineCV).

### 3.3. Genetic Characterisation of the Viruses Identified 

**Figure 1:**
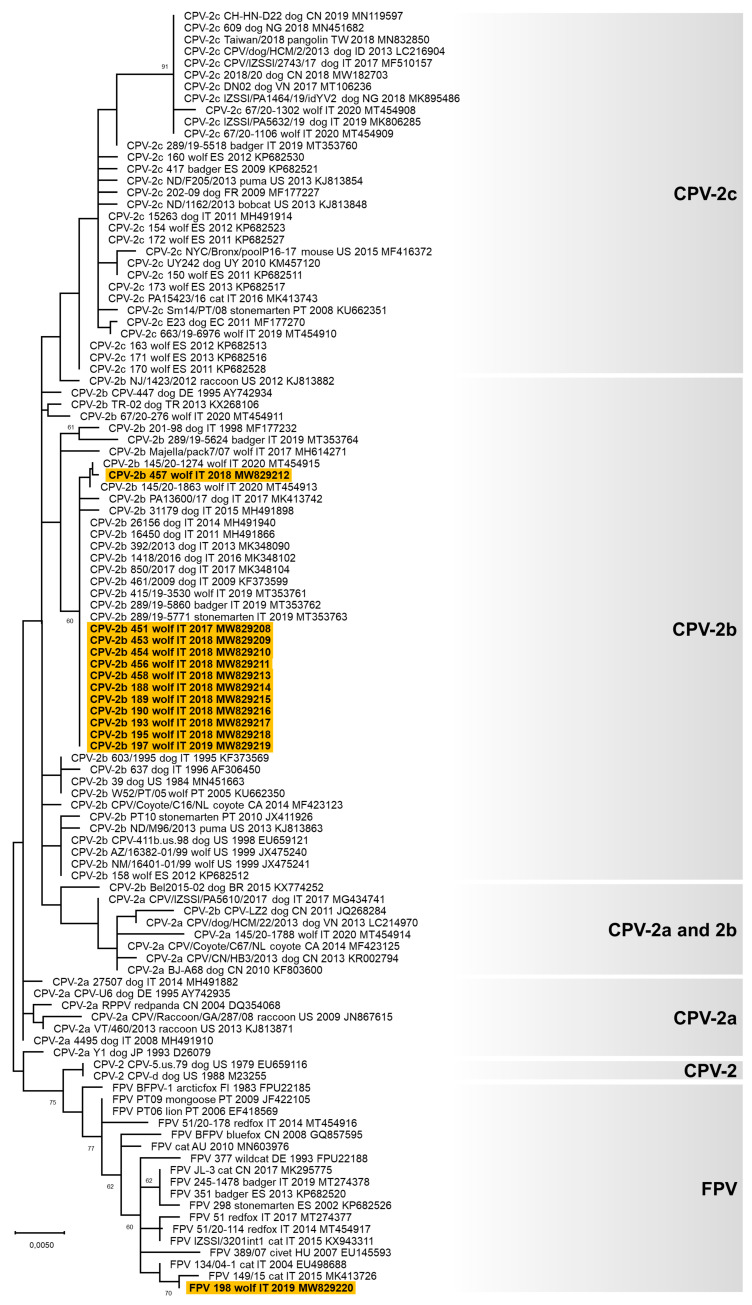
Unrooted phylogenetic tree constructed on the partial VP2 nucleotide sequences of carnivore protoparvoviruses obtained in this study and reference strains in the GenBank database (Table S1), using the Neighbor-Joining method and the Tamura 3-parameters (T92) model with a gamma distribution. The robustness of the individual nodes on the phylogenetic tree was estimated using 1000 bootstrap replicates. Bootstrap values greater than 60% are indicated. The scale bars indicate the estimated numbers of nucleotide substitutions. Highlighted in yellow: sequences of canine parvovirus type 2 (CPV) and feline panleukopenia virus (FPV) generated in this study. In black on the right of the figure are indicated the six groups evidenced in this study.

A partial VP2 gene nucleotide sequence of 532 nts in length (from nucleotide 3669 to nucleotide 4200 of FPV reference strain CU-4, GenBank ID: M38246) was obtained for 13 carnivore protoparvoviruses (GenBank ID: MW829208-MW829220, Table 2 and Table S1). Analysis of the deduced amino acid residues at critical positions allowed the identification of the following carnivore protoparvoviruses [50,55,56,57]: 12 CPV-2b viruses (owing to the occurrence of the amino acid aspartate in position 426) [55] and one FPV-like virus (lab ID: 198/2019). From the nucleotide alignment, all the CPV-2b viruses identified were identical, except for 457/2018 that showed one non-synonymous nucleotide substitution, and all displayed the two distinctive amino acid residues 371-glicine (Gly) and 418-threonine (Thr). Phylogenetic analyses showed that the CPV-2b sequences obtained in this study were closely related to the CPV-2b identified in dogs and wild carnivores from Italy from 2009 to date, while the only FPV-like virus detected was related to FPV identified in cats and wild felids from Europe (Figure 1).

**Figure 2:**
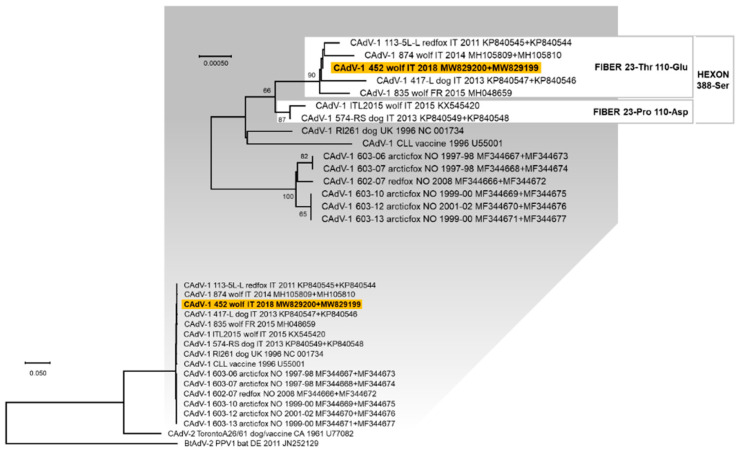
Rooted phylogenetic tree constructed with the multiple gene approach (concatenated nucleotide sequences of the hexon and fiber genes) of canine adenovirus (CAdV) obtained in this study and reference strains in the GenBank database (Table S2). Phylogeny was carried out using the Maximum Likelihood method and Hasegawa–Kishino–Yano (HKY) model with invariable sites. The robustness of the individual nodes on the phylogenetic tree was estimated using 1000 bootstrap replicates. Bootstrap values greater than 60% are indicated. The scale bars indicate the estimated numbers of nucleotide substitutions. On the top of the figure, a portion of the obtained tree is enlarged to better visualise the phylogenetic relationships existing between the CAdV-1 nucleotide sequences and the bootstrap values. For some viruses two GenBank accession numbers are reported (the hexon and fiber genes sequences, respectively). Highlighted in yellow: nucleotide sequence generated in this study. The amino acid residues in position 388 for the deduced hexon protein and in positions 23 and 110 for the deduced fiber protein are reported.

A complete hexon gene nucleotide sequence of 2718 nts in length and a complete fiber gene nucleotide sequence of 1632 nts in length were obtained for one CAdV-1 (lab ID: 452/2017, GenBank ID: MW829199 and MW829200, Table 2 and Table S2). From the concatenated hexon and fiber genes alignment, the identified CAdV-1 452/2017 showed a nucleotide identity of 99.6–99.9% with the reference sequences, whereas the deduced amino acid sequences were identical to reference CAdV-1 identified in dog, red fox, and wolf from Italy and France (113-5L-L: KP840545 and KP840544 [35]; 417-L: KP840547 and KP840546 [53]; and Wolf/835/2015/FRA: MH048659 [27]). In the phylogenetic tree constructed from the concatenated nucleotide sequences of the hexon and fiber genes, the CAdV-1 identified in this study clustered with other CAdV-1 viruses identified in domestic and wild canids from Italy and France, characterised by the distinctive residue 388-serine (Ser) in the deduced hexon protein (Figure 2). This cluster is also divided into two groups: one characterised by the residues 23-proline (Pro) and 110-aspartate (Asp), and the other by the residues 23-threonine (Thr) and 110-glutamate (Glu), in the deduced fiber protein, with CAdV-1 452/2017 belonging to the second one.

**Figure 3:**
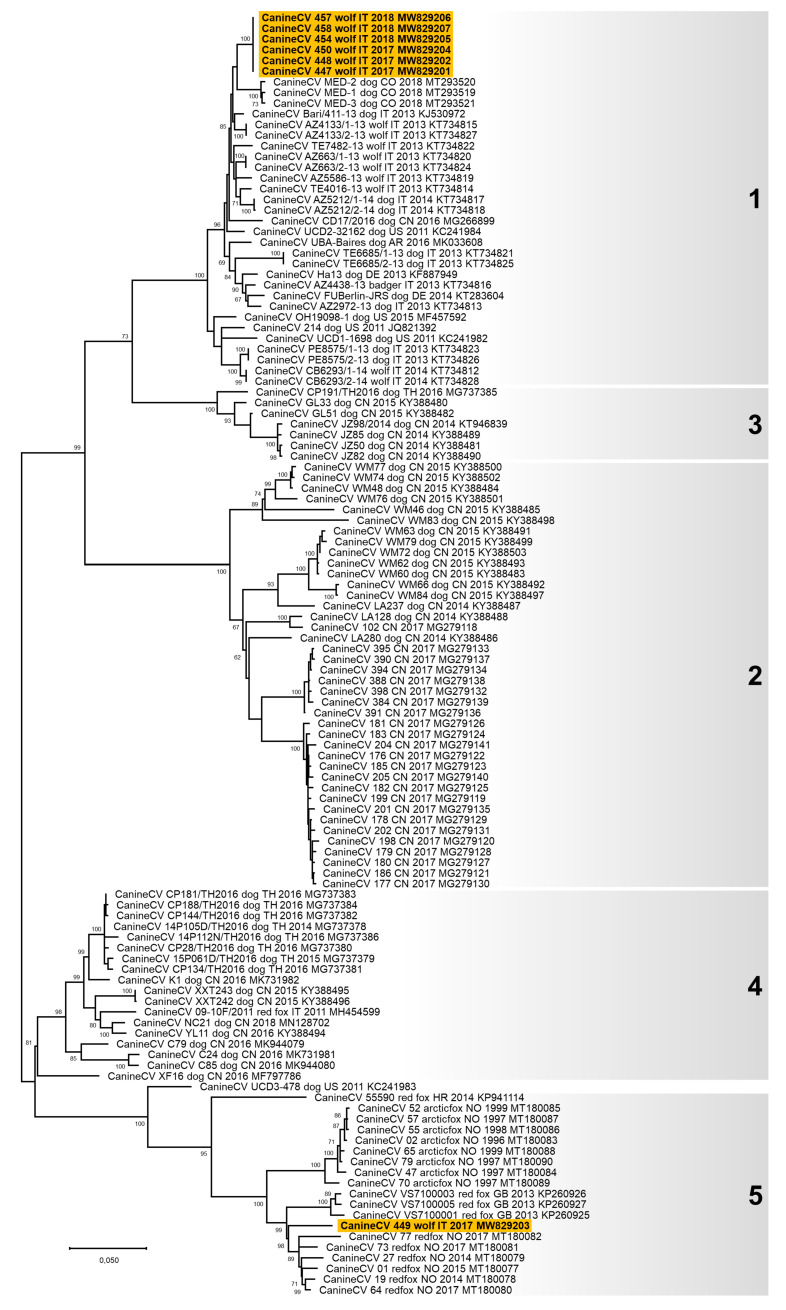
Unrooted phylogenetic tree constructed on the complete genome nucleotide sequences of canine circovirus (CanineCV) obtained in this study and reference strains in the GenBank database (Table S3). Phylogeny was carried out using the Maximum Likelihood method and General Time Reversible (GTR) model with a gamma distribution and invariable sites. The robustness of the individual nodes on the phylogenetic tree was estimated using 1000 bootstrap replicates. Bootstrap values greater than 60% are indicated. The scale bars indicate the estimated number of nucleotide substitutions. Highlighted in yellow: sequences of CanineCV generated in this study. Numbers in black on the right of the figure are the groups evidenced in this study and correspond to the clusters proposed by Niu et al. [60] and Urbani et al. [45].

A complete genome sequence of 2063 nts in length was obtained for seven CanineCVs (GenBank ID: MW829201–MW829207, Table 2 and Table S3). The genome structure was the same as described elsewhere [38,42,45,58,59]. Six viruses had an identical genome and showed a complete genome nucleotide identity of 82.7% with the seventh CanineCV sequenced (lab ID: 449/2017). Nucleotide alignment between the complete genomic sequences of CanineCV obtained in this study and the reference strains showed an overall nucleotide identity ranging from 80.5 to 100%. The phylogenetic tree constructed with the complete genome nucleotide sequences showed a well distinguishable clustering of the CanineCV nucleotide sequences into five groups (Figure 3), as previously proposed by [45,60]. The six CanineCVs with an identical genome identified in this study clustered in Group 1 with the other viruses identified in dogs and wolves worldwide. The CanineCV 449/2017 was included in Group 5 with the viruses identified in foxes in Europe.

## 4. Discussion

Infectious diseases are considered a threat for wild carnivores and the growth of human populations and consequent increase in domestic animals around protected areas enhance the opportunities for disease transmission among species [3]. In this study, all the 23 tested Italian wolves (found dead in the Italian Apennines from 2017 to 2019) were positive for at least one of the DNA viruses screened, and 11/23 (47.8%) were coinfected with two or three viruses, one of which was always a *Carnivore protoparvovirus 1*. Ten out of 23 (43.5%) wolves tested positive for both CPV and CanineCV. A lower frequency (18.7%) of CPV and CanineCV coinfection was described in dogs by Dowgier et al. [40]. Other authors speculated that infection with CPV, which caused crypt epithelial cell and lymphocyte necrosis and subsequent proliferation of regenerating epithelial cells and lymphoblasts, provided the necessary target cells for CanineCV replication and resulted in more severe and prolonged clinical disease [61]. Alternatively, primary infection with CanineCV in dogs may cause immunosuppression and thus allow concurrent or secondary CPV infection [61]. These findings might be true also for wolves. Furthermore, CanineCV might influence the mortality rate in dogs with destruction of the intestinal mucosa barrier, such as in CPV infection [39].

Twenty-one out of 23 (91.3%) wolves tested positive to *Carnivore protoparvovirus 1* DNA. A higher frequency (97.6%) of infection in wolves was reported in Portugal by Rosa et al. [62], while a lower frequency was found in tissue samples of wolves from Spain (67.6%) [16] and Italy (54.3%) [13]. Contrary, other European studies described a much lower frequency of infection using molecular techniques, ranging from 5 to 15.2% in wolves from Portugal and Italy, respectively [18,20]. The higher frequency of *Carnivore protoparvovirus 1* infection found in our study can be explained by several reasons: (i) the higher sensitivity of quantitative PCR compared to conventional PCR and serological techniques performed in other studies [23]; (ii) the use of tongue samples for testing, which has been reported suitable in deceased animals undergoing post-mortem changes [63]; and (iii) the large population of unvaccinated free-ranging dogs present in the Italian Apennines that considerably increases the risk of wild canids exposure to shared infectious pathogens [20]. The identification of carnivore protoparvoviruses in all the Italian regions investigated and in all years of sampling in this study suggests that these viruses are enzootic in Italian wolves, as previously reported in other studies [20,62].

Twelve out of the 13 carnivore protoparvoviruses sequenced for partial VP2 gene were CPV-2b and showed two distinctive amino acid residues: 371-Gly and 418-Thr. CPV-2b with analogous residues in deduced VP2 protein were reported in dogs in Italy from 2008 [52,64,65] and recently in wildlife [13]. This finding supports the hypotheses of an adaptive advantage of these new genotype CPV-2b to spread and stabilise in sensible hosts and of a possible transmission of this virus from domestic to wild carnivores. Interestingly, FPV-like virus DNA was detected in one wolf in the present study. A survey conducted in Italy from 2014 to 2020 did not detect FPV-like viruses in tissue samples from wolves [13], but FPV-like viruses have been detected in red foxes (*Vulpes vulpes*) and Eurasian badgers (*Meles meles*) [13,23]. To the authors’ knowledge, this is the first report describing the detection of an FPV-like virus in wolves, although FPV-like parvoviruses from wild carnivores constitute a transitional form in the evolution of parvoviruses in nature [66]. 

Four out of 23 (17.4%) wolves tested positive for CAdV DNA. Two wolves tested positive to CAdV-1, supporting the possibility that the wolf may transmit CAdV-1 to other wild carnivores or domestic dogs via urine, faeces, and anal gland secretions, representing a risk for not-vaccinated animals [37]. A higher frequency of CAdV-1 infection in wolves were reported in Spain by Oleaga and colleagues (14.1%) [37] and Millán and colleagues (70.3%) [29]. The variable frequency of infection detected can be explained by a genuine non-homogeneous spread of the virus in different geographical areas investigated, as reported for red foxes [67]. The sequenced CAdV-1 (452/2017) phylogenetically grouped with all CAdV-1 viruses from Italy and France that showed the residue 388-Ser in the deduced hexon protein, confirming that this position of the hexon protein is able to differentiate viruses belonging to different geographical regions [25,26,53]. Furthermore, CAdV-1 identified in Italy were distinguishable in two groups on the basis of codons 23 and 110 of the fiber protein, and both have been detected in dogs and wolves. Two wolves tested positive for CAdV-2 DNA in tongue samples, supporting the suitability of this biological matrix for detection of a virus with upper respiratory system tropism. Both wolves tested positive for CAdV-2 were from the Calabria region, confirming the spread of CAdV-2 in wolves from Southern Italy [33], but the low amount of viral DNA detected did not allow the sequencing of the viruses identified. CAdV-2 infection was also reported in wolves from Northern Italy and Spain [29,34], but its real spread and pathogenic role are currently unknown.

Eleven out of 23 (47.8%) wolves tested positive for CanineCV DNA. The only study carried out on this pathogen in wolves showed a lower frequency of infection (26.4%) in Italy [19]. These findings confirm that CanineCV infection is not restricted to dogs [39,40,44] but is also widespread in wild animals [19,41,45,46]. From the complete viral genome analysis, the CanineCV identified and sequenced in this study belonged to a unique viral species infecting dogs, foxes, wolves, and badgers, showing a nucleotide identity >80%, representing the species demarcation threshold of the genome-wide nucleotide sequence identity for members of the family *Circoviridae* [68,69]. All the CanineCVs identified and sequenced in this study, with the exception of CanineCV 449/2017, were strictly related to viruses identified in dogs and wolves worldwide, supporting the hypothesis of a possible transmission of circovirus infection between domestic and wild carnivores, but the pathogenetic role of this virus in wildlife is still not clear and further investigation is needed. Differently, CanineCV 449/2017 (GenBank ID: MW829203) grouped within viruses only identified in foxes, disproving the clustering of all fox-infecting CanineCV into a single group [19], and confirming the absence of a strict species-specificity for fox-infecting CanineCV, previously questioned by the detection in a red fox of a virus genetically related to CanineCV identified in dogs [46].

The present study has some limitations. A small number of wolves were included, limiting the representativeness of the results obtained. This is due to the sampling difficulties of free-ranging protected animal species, mainly the impossibility of collecting all the carcasses of deceased subjects. Furthermore, only DNA viruses were screened in our population. Thus, coinfections with RNA viruses (such as canine coronavirus or canine distemper virus) were not investigated. The screening was targeted only towards DNA viruses considering the storage conditions of the tested organs, as the state of decomposition of some subjects could have determined the degradation of the RNA, altering the reliability of the result.

## 5. Conclusions

In this study, new data on the presence of DNA viruses and viral coinfection in wolves in Italy are reported, suggesting an active viral circulation among the wolf population from Italy. The genetic characterization of the identified viruses suggests that the same viruses infect both the wolf and the domestic dog. Further studies are needed to investigate if these pathogens are transmitted between the two species. The high frequency of detection and the variety of viral pathogens identified confirms the epidemiological role of wolves as hosts of viral infectious agents, as well as the pathogen pressure on the health status of wild animals. Therefore, the data obtained underline that wild carnivores can serve as reservoirs for infectious agents and the importance of domestic dog vaccination to reduce the potential transmission of pathogens to wildlife and vice versa.

## Figures and Tables

**Table 1:** Wolves included in this study and tested for *Carnivore protoparvovirus 1*, Canine adenovirus (CAdV), and Canine circovirus (CanineCV) DNA.

Variables	Total	*Carnivore protoparvovirus 1*	CAdV	CanineCV	Coinfections
Number of wolves	23	21 (91.3)	4 (17.4)	11 (47.8)	11 (47.8)
Sex					
Male	14 (60.9)	13	4	7	8
Female	9 (39.1)	8	0	4	3
Age (months)	24 (3–48)	24 (5–48)	30 (12–36)	24 (3–48)	24 (5–48)
Puppies (≤12)	10 (43.5)	8	1	5	4
Sub-adults (13–24)	7 (30.4)	7	1	3	3
Adults (>24)	6 (26.1)	6	2	3	4
Geographical origin					
Emilia Romagna	13 (56.5)	12	1	8	7
Tuscany	8 (34.8)	7	1	2	2
Calabria	2 (8.7)	2	2	1	2
Year of sampling					
2017	6 (26.1)	4	1	5	4
2018	13 (56.5)	13	2	6	6
2019	4 (17.4)	4	1	0	1

Data are expressed as the number of wolves (percentage in parentheses) or as the median (range in parentheses).

**Table 2:** Results of the *Carnivore protoparvovirus 1*, Canine adenovirus type 1 and 2 (CAdV-1 and CAdV-2), and Canine circovirus (CanineCV) DNA screening.

Wolf	*Carnivore protoparvovirus 1*			CAdV-1		CAdV-2		CanineCV	
	Tongue	Intestine	Tongue		Spleen	Tongue	Spleen	Intestine	Spleen
447/2017	-	-	-		-	-	-	P	P S
448/2017	P	-	-		-	-	-	-	P S
449/2017	-	P	-		-	-	-	P	P S
450/2017	P	P	-		-	-	-	P S	P
451/2017	P S	P	-		-	-	-	P	P
452/2017	-	-	-		P S	-	-	-	-
453/2018	P S	-	-		-	-	-	-	-
454/2018	P	P S	-		-	P	-	P S	P
455/2018	P	P	-		-	-	-	-	P
456/2018	-	P S	-		-	-	-	P	-
457/2018	P	P S	-		-	-	-	P S	P
458/2018	P	P S	-		-	-	-	P S	P
188/2018	P S	P	-		-	-	-	-	-
189/2018	P S	P	-		-	-	-	-	-
190/2018	-	P S	-		P	-	-	-	P
191/2018	P	-	-		-	-	-	-	-
193/2018	-	P S	-		-	-	-	-	-
194/2019	P	-	-		-	-	-	-	-
195/2018	P	P S	-		-	-	-	-	-
196/2018	-	P	-		-	-	-	-	-
197/2019	P	P S	-		-	-	-	-	-
198/2019	P S	-	-		-	P	-	-	-
199/2019	P	P	-		-	-	-	-	-

CAdV-1: Canine adenovirus type 1; CAdV-2: Canine adenovirus type 2; CanineCV: Canine circovirus; P: positive; -: negative; S: viruses sequenced.

## Data Availability

The datasets supporting the conclusions of this article are included within the article (and its supplementary files). The viral nucleotide sequences obtained in this study are openly available in GenBank database (https://www.ncbi.nlm.nih.gov/genbank/, accessed 12 on April 2021; ID: MW829199-MW829220).

## Supplementary Materials

The following are available online at https://www.mdpi.com/article/10.3390/ani11082198/s1. Table S1: *Carnivore protoparvovirus 1* nucleotide sequences obtained in this study and reference strains retrieved from GenBank used for analysis, Table S2: Canine adenovirus nucleotide sequences obtained in this study and reference strains retrieved from GenBank used for analysis, Table S3: Canine circovirus nucleotide sequences obtained in this study and reference strains retrieved from GenBank used for analysis.
