# Supplementary material for: Integrated Use of Molecular Techniques to Detect and Genetically Characterise DNA Viruses in Italian Wolves (Canis lupus italicus)
Source: Animals (Basel). 2021 Jul 24;11(8):2198. doi: 10.3390/ani11082198 (PMC8388400; doi:10.3390/ani11082198)
Supplement: Supplementary file 1 [file animals-11-02198-s001.zip › 20210615 TableS1.pdf]

Table S1. *Carnivore protoparvovirus 1* nucleotide sequences obtained in this study and reference strains retrieved from GenBank used for analysis.

GenBank ID	Strain	Pathogen	Genome size (nt)	Host	Sample	Year	Origin
AF306450	637	CPV-2b	1745	Dog	NA	1996	Italy
AY742934	CPV-447	CPV-2b	4432	Dog	NA	1995	Germany
AY742935	CPV-U6	CPV-2a	4432	Dog	NA	1995	Germany
D26079	Y1	CPV-2a	5075	Dog	NA	1993	Japan
DQ354068	RPPV	CPV-2a	1755	Red panda	NA	2004	China
EF418569	PT06	FPV	1850	Lion	Faeces	2006	Portugal
EU145593	389/07	FPV	1755	Asian palm civet	NA	2007	Hungary
EU498688	134/04-1	FPV	1755	Cat	Faeces	2004	Italy
EU659116	CPV-5.us.79	CPV-2	4269	Dog	NA	1979	USA
EU659121	CPV-411b.us.98	CPV-2b	4269	Dog	NA	1998	USA
FPU22185	BFPV-1	FPV	2256	Arctic fox	NA	1983	Finland
FPU22188	377	FPV	1957	Wildcat	NA	1993	Germany
GQ857595	BFPV	FPV	1755	Blue fox	NA	2008	China
JF422105	PT09	FPV	1755	Egyptian mongoose	Intestine, lymph node	2009	Portugal
JN867615	CPV/Raccoon/GA/287/08	CPV-2a	4627	Raccoon	NA	2009	USA
JQ268284	CPV-LZ2	CPV-2b	5053	Dog	NA	2011	China
JX411926	PT10	CPV-2b	1755	Stone marten	Intestine, lymph node	2010	Portugal
JX475240	AZ/16382-01/99	CPV-2b	1755	Wolf	NA	1999	USA
JX475241	NM/16401-01/99	CPV-2b	1755	Wolf	NA	1999	USA
KF373569	603/1995	CPV-2b	1745	Dog	NA	1995	Italy
KF373599	461/2009	CPV-2b	1745	Dog	NA	2009	Italy
KF803600	BJ-A68	CPV-2a	1752	Dog	NA	2010	China
KJ813848	ND/1162/2013	CPV-2c	1755	Bobcat	NA	2013	USA
KJ813854	ND/F205/2013	CPV-2c	1755	Puma	NA	2013	USA
KJ813863	ND/M96/2013	CPV-2b	1755	Puma	NA	2013	USA
KJ813871	VT/460/2013	CPV-2a	1755	Raccoon	NA	2013	USA
KJ813882	NJ/1423/2012	CPV-2b	1755	Raccoon	NA	2012	USA
KM457120	UY242	CPV-2c	4269	Dog	NA	2010	Uruguay
KP682511	150	CPV-2c	1746	Wolf	Spleen	2011	Spain
KP682512	158	CPV-2b	1746	Wolf	Spleen	2012	Spain
KP682513	163	CPV-2c	1746	Wolf	Spleen	2012	Spain

KP682516	171	CPV-2c	1746	Wolf	Spleen	2013	Spain
KP682517	173	CPV-2c	1746	Wolf	Spleen	2013	Spain
KP682520	351	FPV	1746	Badger	Spleen	2013	Spain
KP682521	417	CPV-2c	1746	Badger	Spleen	2009	Spain
KP682523	154	CPV-2c	1746	Wolf	Spleen	2012	Spain
KP682526	298	FPV	1746	Stone marten	Spleen	2002	Spain
KP682527	172	CPV-2c	1746	Wolf	Spleen	2011	Spain
KP682528	170	CPV-2c	1746	Wolf	Spleen	2011	Spain
KP682530	160	CPV-2c	1678	Wolf	Spleen	2012	Spain
KR002794	CPV/CN/HB3/2013	CPV-2a	4269	Dog	NA	2013	China
KU662350	W52/PT/05	CPV-2b	1755	Wolf	Liver	2005	Portugal
KU662351	Sm14/PT/08	CPV-2c	1755	Stone marten	Spleen	2008	Portugal
KX268106	TR-02	CPV-2b	1752	Dog	NA	2013	Turkey
KX774252	Bel2015-02	CPV-2b	5020	Dog	NA	2015	Brazil
KX943311	IZSSI/3201int1	FPV	1745	Cat	Intestine	2015	Italy
LC214970	CPV/dog/HCM/22/2013	CPV-2a	4269	Dog	NA	2013	Vietnam
LC216904	CPV/dog/HCM/2/2013	CPV-2c	1755	Dog	NA	2013	Indonesia
M23255	CPV-d	CPV-2	2605	Dog	NA	1988	USA
MF177227	202-09	CPV-2c	4269	Dog	NA	2009	France
MF177232	201-98	CPV-2b	4269	Dog	NA	1998	Italy
MF177270	E23	CPV-2c	4269	Dog	NA	2011	Ecuador
MF416372	NYC/Bronx/poolP16-17	CPV-2c	4809	Mouse	Faeces	2015	USA
MF423123	CPV/Coyote/C16/NL	CPV-2b	4468	Coyote	NA	2014	Canada
MF423125	CPV/Coyote/C67/NL	CPV-2a	4468	Coyote	NA	2014	Canada
MF510157	CPV/IZSSI/2743/17	CPV-2c	4450	Dog	Intestine	2017	Italy
MG434741	CPV/IZSSI/PA5610/2017	CPV-2a	4381	Dog	Rectal swab	2017	Italy
MH491866	16450	CPV-2b	1752	Dog	NA	2011	Italy
MH491882	27507	CPV-2a	1752	Dog	NA	2014	Italy
MH491898	31179	CPV-2b	1752	Dog	NA	2015	Italy
MH491910	4495	CPV-2a	1752	Dog	NA	2008	Italy
MH491914	15263	CPV-2c	1752	Dog	NA	2011	Italy
MH491940	26156	CPV-2b	1737	Dog	NA	2014	Italy
MH614271	Majella/pack7/07	CPV-2b	1752	Wolf	Faeces	2017	Italy
MK295775	JL-3	FPV	1755	Cat	NA	2017	China
MK348090	392/2013	CPV-2b	1755	Dog	Faeces	2013	Italy
MK348102	1418/2016	CPV-2b	1755	Dog	Faeces	2016	Italy
MK348104	850/2017	CPV-2b	1755	Dog	Faeces	2017	Italy

MK413726	149/15	FPV	4269	Cat	Intestine	2015	Italy
MK413742	PA13600/17	CPV-2b	4269	Dog	Spleen	2017	Italy
MK413743	PA15423/16	CPV-2c	4269	Cat	Spleen	2016	Italy
MK806285	IZSSI/PA5632/19	CPV-2c	4269	Dog	Spleen	2019	Italy
MK895486	IZSSI/PA1464/19/idYV2	CPV-2c	4269	Dog	Rectal swab	2018	Nigeria
MN119597	CH-HN-D22	CPV-2c	1755	Dog	NA	2019	China
MN451663	39	CPV-2b	4269	Dog	Faeces	1984	USA
MN451682	609	CPV-2c	4269	Dog	Faeces	2018	Nigeria
MN603976	Tasman island/Tasmania/Felis catus/2010	FPV	1744	Cat	NA	2010	Australia
MN832850	Taiwan/2018	CPV-2c	4960	Pangolin	NA	2018	Taiwan
MT106236	DN02	CPV-2c	4269	Dog	NA	2017	Vietnam
MT274377	51	FPV	1755	Red fox	NA	2017	Italy
MT274378	245/19-1478	FPV	1755	Badger	Intestine	2019	Italy
MT353760	289/19-5518	CPV-2c	1755	Badger	Intestine	2019	Italy
MT353761	415/19-3530	CPV-2b	1755	Wolf	Intestine	2019	Italy
MT353762	289/19-5860	CPV-2b	1755	Badger	Intestine	2019	Italy
MT353763	289/19-5771	CPV-2b	1755	Stone marten	Intestine	2019	Italy
MT353764	289/19-5624	CPV-2b	1755	Badger	Intestine	2019	Italy
MT454908	67/20-1302	CPV-2c	736	Wolf	Intestine	2020	Italy
MT454909	67/20-1106	CPV-2c	736	Wolf	Intestine	2020	Italy
MT454910	663/19-6976	CPV-2c	736	Wolf	NA	2020	Italy
MT454911	67/20-276	CPV-2b	736	Wolf	Intestine	2020	Italy
MT454913	145/20-1863	CPV-2b	736	Wolf	Intestine	2020	Italy
MT454914	145/20-1788	CPV-2a	736	Wolf	Intestine	2020	Italy
MT454915	145/20-1274	CPV-2b	736	Wolf	Intestine	2020	Italy
MT454916	51/20-178	FPV	736	Red fox	Spleen	2014	Italy
MT454917	51/20-114	FPV	736	Red fox	NA	2014	Italy
MW182703	2018/20	CPV-2c	1755	Dog	NA	2018	China
**MW829208**	**451**	**CPV-2b**	**532**	**Wolf**	**Tongue**	**2017**	**Italy**
**MW829209**	**453**	**CPV-2b**	**532**	**Wolf**	**Tongue**	**2018**	**Italy**
**MW829210**	**454**	**CPV-2b**	**532**	**Wolf**	**Intestine**	**2018**	**Italy**
**MW829211**	**456**	**CPV-2b**	**532**	**Wolf**	**Intestine**	**2018**	**Italy**
**MW829212**	**457**	**CPV-2b**	**532**	**Wolf**	**Intestine**	**2018**	**Italy**
**MW829213**	**458**	**CPV-2b**	**532**	**Wolf**	**Intestine**	**2018**	**Italy**
**MW829214**	**188**	**CPV-2b**	**532**	**Wolf**	**Tongue**	**2018**	**Italy**
**MW829215**	**189**	**CPV-2b**	**532**	**Wolf**	**Tongue**	**2018**	**Italy**
**MW829216**	**190**	**CPV-2b**	**532**	**Wolf**	**Intestine**	**2018**	**Italy**

**MW829217**	**193**	**CPV-2b**	**532**	**Wolf**	**Intestine**	**2018**	**Italy**
**MW829218**	**195**	**CPV-2b**	**532**	**Wolf**	**Intestine**	**2018**	**Italy**
**MW829219**	**197**	**CPV-2b**	**532**	**Wolf**	**Intestine**	**2019**	**Italy**
**MW829220**	**198**	**FPV**	**532**	**Wolf**	**Tongue**	**2019**	**Italy**

Note: CPV = canine parvovirus type 2, CPV-2a = canine parvovirus 2a, CPV-2b = canine parvovirus 2b, CPV-2c = canine parvovirus 2c, FPV = feline panleukopenia virus, NA = not available, nt = nucleotides.

In bold: *Carnivore protoparvovirus 1* sequences obtained in this study.
