# Supplementary material for: Integrated Use of Molecular Techniques to Detect and Genetically Characterise DNA Viruses in Italian Wolves (Canis lupus italicus)
Source: Animals (Basel). 2021 Jul 24;11(8):2198. doi: 10.3390/ani11082198 (PMC8388400; doi:10.3390/ani11082198)
Supplement: Supplementary file 1 [file animals-11-02198-s001.zip › 20210615 TableS2.pdf]

Table S2. Canine adenovirus nucleotide sequences obtained in this study and reference strains retrieved from GenBank used for analysis.

GenBank ID	Strain	Pathogen	Sequence size (nts)	Host	Sample	Year	Origin
JN252129	PPV1	BtAdV	31616	Bat	Liver/kidney	2011	Germany
KP840545 + KP840544	113-5L	CAdV-1	2718 + 1632	Red fox	Liver	2011	Italy
KP840547 + KP840546	417-L	CAdV-1	2718 + 1632	Dog	Liver	2013	Italy
KP840549 + KP840548	574-RS	CAdV-1	2718 + 1632	Dog	Rectal swab	2013	Italy
KX545420	ITL2015	CAdV-1	30531	Wolf	Liver	2015	Italy
MF344666 + MF344672	602-07	CAdV-1	2718 + 1632	Red fox	Spleen	2008	Norway
MF344667 + MF344673	603-06	CAdV-1	2718 + 1632	Arctic fox	Liver	1997-98	Norway
MF344668 + MF344674	603-07	CAdV-1	2718 + 1632	Arctic fox	Kidney	1997-98	Norway
MF344669 + MF344675	603-10	CAdV-1	2718 + 1632	Arctic fox	Spleen	1999-00	Norway
MF344670 + MF344676	603-12	CAdV-1	2718 + 1632	Arctic fox	Spleen	2001-02	Norway
MF344671 + MF344677	603-13	CAdV-1	2718 + 1632	Arctic fox	Lymph node	1999-00	Norway
MH048659	835	CAdV-1	30534	Wolf	Liver	2015	France
MH105809 + MH105810	874	CAdV-1	2718 + 1632	Wolf	Tongue	2014	Italy
**MW829200 + MW829199**	**452**	**CAdV-1**	**2718 + 1632**	**Wolf**	**Spleen**	**2017**	**Italy**
NC_001734	RI261	CAdV-1	30536	Dog	NA	1996	Great Britain
U55001	CLL	CAdV-1	30288	Dog/vaccine	Kidney	1996	NA
U77082	TorontoA26/61	CAdV-2	31323	Dog/vaccine	NA	1961	Canada

Note: NA = not available, nt = nucleotides.

In bold: Canine adenovirus sequences obtained in this study.
