# Supplementary material for: Integrated Use of Molecular Techniques to Detect and Genetically Characterise DNA Viruses in Italian Wolves (Canis lupus italicus)
Source: Animals (Basel). 2021 Jul 24;11(8):2198. doi: 10.3390/ani11082198 (PMC8388400; doi:10.3390/ani11082198)
Supplement: Supplementary file 1 [file animals-11-02198-s001.zip › 20210615 TableS3.pdf]

Table S3. Canine circovirus nucleotide sequences obtained in this study and reference strains retrieved from GenBank used for analysis.

GenBank ID	Strain	Genome size (nt)	Host	Sample	Year	Origin	I	P
JQ821392	214	2063	Dog	Serum	2011	USA		
KC241982 (NC020904)	UCD1-1698	2063	Dog	Liver	2011	USA		
KC241983	UCD3-478	2063	Dog	Blood	2011	USA		
KC241984	UCD2-32162	2063	Dog	Faeces	2011	USA		
KF887949	Ha13	2063	Dog	Blood	2013	Germany		
KJ530972	Bari/411-13	2063	Dog	Intestine	2013	Italy		
KP260925	VS7100001	2063	Red fox	Serum	2013	United Kingdom		
KP260926	VS7100003	2063	Red fox	Serum	2013	United Kingdom		
KP260927	VS7100005	2063	Red fox	Serum	2013	United Kingdom		
KP941114	55590	2055	Red fox	Faeces	2014	Croatia		
KT283604	FUBerlin-JRS	2063	Dog	Spleen	2014	Germany		
KT734812	CB6293/1-14	2063	Wolf	Mesenteric lymph node	2014	Italy		
KT734813	AZ2972-13	2063	Dog	Intestine	2013	Italy		
KT734814	TE4016-13	2063	Wolf	Spleen	2013	Italy		
KT734815	AZ4133/1-13	2063	Wolf	Spleen	2013	Italy		
KT734816	AZ4438-13	2063	Badger	Intestine	2013	Italy		
KT734817	AZ5212/1-14	2063	Dog	Liver	2014	Italy		
KT734818	AZ5212/2-14	2063	Dog	Lung	2014	Italy		
KT734819	AZ5586-13	2063	Wolf	Lung	2013	Italy		
KT734820	AZ663/1-13	2063	Wolf	Spleen	2013	Italy		
KT734821	TE6685/1-13	2063	Dog	Brain	2013	Italy		
KT734822	TE7482-13	2063	Wolf	Intestine	2013	Italy		
KT734823	PE8575/1-13	2063	Dog	Mesenteric lymph node	2013	Italy		
KT734824	AZ663/2-13	2063	Wolf	Intestine	2013	Italy		
KT734825	TE6685/2-13	2063	Dog	Lung	2013	Italy		
KT734826	PE8575/2-13	2063	Dog	Intestine	2013	Italy		
KT734827	AZ4133/2-13	2063	Wolf	Intestine	2013	Italy		
KT734828	CB6293/2-14	2063	Wolf	Intestine	2014	Italy		
KT946839	JZ98/2014	2063	Dog	Serum	2014	China		
KY388480	GL33	2063	Dog	Serum	2015	China		
KY388481	JZ50	2063	Dog	Serum	2014	China		
KY388482	GL51	2063	Dog	Serum	2015	China		

KY388483	WM60	2064	Dog	Serum	2015	China
KY388484	WM48	2064	Dog	Serum	2015	China
KY388485	WM46	2064	Dog	Serum	2015	China
KY388486	LA280	2063	Dog	Serum	2014	China
KY388487	LA237	2064	Dog	Serum	2014	China
KY388488	LA128	2063	Dog	Serum	2014	China
KY388489	JZ85	2063	Dog	Serum	2014	China
KY388490	JZ82	2063	Dog	Serum	2014	China
KY388491	WM63	2064	Dog	Serum	2015	China
KY388492	WM66	2064	Dog	Serum	2015	China
KY388493	WM62	2064	Dog	Serum	2015	China
KY388494	YL11	2063	Dog	Serum	2016	China
KY388495	XXT243	2063	Dog	Serum	2015	China
KY388496	XXT242	2063	Dog	Serum	2015	China
KY388497	WM84	2064	Dog	Serum	2015	China
KY388498	WM83	2063	Dog	Serum	2015	China
KY388499	WM79	2064	Dog	Serum	2015	China
KY388500	WM77	2064	Dog	Serum	2015	China
KY388501	WM76	2064	Dog	Serum	2015	China
KY388502	WM74	2064	Dog	Serum	2015	China
KY388503	WM72	2064	Dog	Serum	2015	China
MF457592	OH19098-1	2063	Dog	Spleen	2015	USA
MF797786	XF16	2063	Dog	Faeces	2016	China
MG266899	CD17/2016	2063	Dog	Faeces	2016	China
MG279118	102	2063	NA	NA	2017	China
MG279119	199	2063	NA	NA	2017	China
MG279120	198	2063	NA	NA	2017	China
MG279121	186	2063	NA	NA	2017	China
MG279122	176	2063	NA	NA	2017	China
MG279123	185	2063	NA	NA	2017	China
MG279124	183	2063	NA	NA	2017	China
MG279125	182	2063	NA	NA	2017	China
MG279126	181	2063	NA	NA	2017	China
MG279127	180	2063	NA	NA	2017	China
MG279128	179	2063	NA	NA	2017	China
MG279129	178	2063	NA	NA	2017	China
MG279130	177	2063	NA	NA	2017	China

MG279131	202	2063	NA	NA	2017	China
MG279132	398	2064	NA	NA	2017	China
MG279133	395	2064	NA	NA	2017	China
MG279134	394	2064	NA	NA	2017	China
MG279135	201	2063	NA	NA	2017	China
MG279136	391	2064	NA	NA	2017	China
MG279137	390	2064	NA	NA	2017	China
MG279138	388	2064	NA	NA	2017	China
MG279139	384	2064	NA	NA	2017	China
MG279140	205	2063	NA	NA	2017	China
MG279141	204	2063	NA	NA	2017	China
MG737378	14P105D/TH2016	2039	Dog	Lung	2014	Thailand
MG737379	15P061D/TH2016	1911	Dog	Lung	2015	Thailand
MG737380	CP28/TH2016	1907	Dog	Nasal swab	2016	Thailand
MG737381	CP134/TH2016	1902	Dog	Nasal swab	2016	Thailand
MG737382	CP144/TH2016	1905	Dog	Nasal swab	2016	Thailand
MG737383	CP181/TH2016	1907	Dog	Nasal swab	2016	Thailand
MG737384	CP188/TH2016	1903	Dog	Nasal swab	2016	Thailand
MG737385	CP191/TH2016	1954	Dog	Nasal swab	2016	Thailand
MG737386	14P112N/TH2016	1989	Dog	Lung	2016	Thailand
MH454599	09-10F/2011	2001	Red fox	Faeces	2011	Italy
MK033608	UBA-Baires	2063	Dog	Lymph node	2016	Argentina
MK731981	C24	2063	Dog	Faeces	2016	China
MK731982	K1	2063	Dog	Faeces	2016	China
MK944079	C79	2063	Dog	Faeces	2016	China
MK944080	C85	2063	Dog	Faeces	2016	China
MN128702	NC21	2063	Dog	NA	2018	China
MT180077	gen-15	2063	Red fox	Spleen	2015	Norway
MT180078	19/2014	2063	Red fox	Spleen	2014	Norway
MT180079	27/2014	2063	Red fox	Spleen	2014	Norway
MT180080	64/2017	2063	Red fox	Liver	2017	Norway
MT180081	73/2017	2063	Red fox	Spleen	2017	Norway
MT180082	77/2017	2063	Red fox	Spleen	2017	Norway
MT180083	feb-96	2063	Arctic fox	Liver	1996	Norway
MT180084	47/1997	2063	Arctic fox	Spleen	1997	Norway
MT180085	52/1999	2063	Arctic fox	Spleen	1999	Norway
MT180086	55/1998	2063	Arctic fox	Spleen	1998	Norway

MT180087	57/1997	2063	Arctic fox	Spleen	1997	Norway	
MT180088	65/1999	2063	Arctic fox	Liver	1999	Norway	
MT180089	70/1997	2063	Arctic fox	Liver	1997	Norway	
MT180090	79/1997	2063	Arctic fox	Liver	1997	Norway	
MT293519	MED-1	2063	Dog	Faeces	2018	Colombia	
MT293520	MED-2	2063	Dog	Faeces	2018	Colombia	
MT293521	MED-3	2063	Dog	Faeces	2018	Colombia	
**MW829201**	**447**	**2063**	**Wolf**	**Spleen**	**2017**	**Italy**	
**MW829202**	**448**	**2063**	**Wolf**	**Spleen**	**2017**	**Italy**	
**MW829203**	**449**	**2063**	**Wolf**	**Spleen**	**2017**	**Italy**	
**MW829204**	**450**	**2063**	**Wolf**	**Intestine**	**2017**	**Italy**	
**MW829205**	**454**	**2063**	**Wolf**	**Intestine**	**2018**	**Italy**	
**MW829206**	**457**	**2063**	**Wolf**	**Intestine**	**2018**	**Italy**	
**MW829207**	**458**	**2063**	**Wolf**	**Intestine**	**2018**	**Italy**	

Note: I = sequences used for nucleotide identity, NA = not available, nt = nucleotides, P = sequences used for phylogenetic analysis.

In bold: Canine circovirus sequences obtained in this study.
